# Manipulation of Epithelial Differentiation by HPV Oncoproteins

**DOI:** 10.3390/v11040369

**Published:** 2019-04-22

**Authors:** Elizabeth A. White

**Affiliations:** 1 Department of Otorhinolaryngology: Head and Neck Surgery, University of Pennsylvania Perelman School of Medicine, Philadelphia, PA 19104, USA; eawhite@pennmedicine.upenn.edu; 2 Department of Microbiology, University of Pennsylvania Perelman School of Medicine, Philadelphia, PA 19104, USA

**Keywords:** virus, transformation, epithelial, differentiation, HPV

## Abstract

Papillomaviruses replicate and cause disease in stratified squamous epithelia. Epithelial differentiation is essential for the progression of papillomavirus replication, but differentiation is also impaired by papillomavirus-encoded proteins. The papillomavirus E6 and E7 oncoproteins partially inhibit and/or delay epithelial differentiation and some of the mechanisms by which they do so are beginning to be defined. This review will outline the key features of the relationship between HPV infection and differentiation and will summarize the data indicating that papillomaviruses alter epithelial differentiation. It will describe what is known so far and will highlight open questions about the differentiation-inhibitory mechanisms employed by the papillomaviruses.

## 1. Introduction

Papillomaviruses and their host species have evolved together for tens of millions of years, and it is thought that the viruses have both adapted to specific host niches and co-speciated with their hosts [1]. Papillomavirus infections are highly specific for an individual vertebrate species and restricted to a specific tissue: the stratified squamous epithelium [2]. A highly regulated differentiation program enables stratified epithelia to constantly self-renew and papillomavirus replication is enabled by epithelial differentiation [3,4]. Differentiation is required for replication of the papillomavirus DNA genome and for late viral gene expression, and the progression of epithelial cells from basal layers to eventual desquamation enables papillomavirus assembly and egress. Papillomavirus replication requires epithelial differentiation, but also delays it. To enable viral genome replication in infected epithelial cells that would have otherwise committed to a terminal differentiation program, the virus must promote proliferation in the differentiating cell. This is said to ‘uncouple’ proliferation from differentiation. Papillomaviruses inhibit differentiation via a variety of mechanisms, some of which overlap with the pro-proliferative targets and others that are distinct.

The human papillomavirus (HPV) E6 and E7 proteins have been the most frequently characterized with respect to their effects on cellular proliferation and, to some degree, on differentiation. Some of the pro-proliferative mechanisms employed by HPV E6 and E7 are shared by other DNA tumor viruses that do not specifically infect stratified epithelia. For example, several DNA tumor viruses inactivate the retinoblastoma and p53 tumor suppressors to promote proliferation and evade apoptosis [5]. Other viral activities are unique to the papillomavirus-encoded proteins, and these unique activities have allowed the adaptation of papillomaviruses to their specific species and to specific biological niches [1]. The ability to control epithelial differentiation is one such specific activity. HPVs have been recognized to alter differentiation for many years, but recent advances have begun to clarify the mechanisms by which they do so.

This review will summarize the studies that have established the effects of HPV-encoded proteins, particularly HPV E6 and E7, on epithelial differentiation. It will highlight some of the mechanisms by which HPV E6 and E7 delay and impair differentiation. Impaired differentiation is a hallmark of some cancers and could contribute to HPV-mediated carcinogenesis. Open questions related to HPV control of epithelial differentiation and the possibility of differentiation therapy for HPV-positive cancers will be discussed.

## 2. Differentiation-Dependent HPV Biology

HPV have circular double-stranded DNA genomes of ~8 kbp that encode seven to nine open reading frames (ORF) depending on the specific virus. Genetic diversity among the papillomaviruses (PV) is high. Well over 200 HPV genotypes and hundreds more animal PV genotypes have been identified and sequenced and many more are pending characterization [6,7]. Individual PV genotypes differ from one another by at least 10% at the DNA sequence level and they are commonly arranged into a phylogeny based upon the sequence of the L1 gene [2]. The known HPV group into five genera (alpha, beta, gamma, mu, and nu) in an L1-based phylogenetic tree and the majority of the HPV are in genus alpha, beta, or gamma. The genera are further subdivided into species on the basis of sequence similarity. PV that differ by less than 10% are referred to as subtypes and variants. Genus alpha HPV are predominately, but not exclusively, mucosal-tropic, whereas the genus beta and genus gamma HPV infect cutaneous epithelia.

Several features of the productive HPV life cycle depend on the biology of the stratified squamous epithelium. Upon initial infection, HPV particles bind to heparan sulfate proteoglycans present on the cell membrane or in the extracellular matrix [8,9]. A subsequent conformational change in L1 and L2 capsid proteins enables their cleavage by furin or other host enzymes. This enables virus entry into basal epithelial cells via a slow process involving endocytosis and uptake into endosomes. The L2 protein is required to deliver the viral genome to the nucleus in a process recently shown to require a cell-penetrating peptide (CPP) sequence conserved in L2 [10]. The ability of L2 to penetrate the endosomal membrane and to bind to the retromer complex allows for trafficking of the viral particle within the endosome.

Following deposition of the HPV DNA in the nucleus, a burst of viral genome amplification establishes HPV genomes in basal epithelial cells. Viral DNA replication requires the HPV E1 and E2 proteins as well as components of the host cell DNA replication machinery [2,11]. In a productive infection, viral genomes are maintained over the lifetime of the infected cell, replicating once per cell cycle. Maintenance replication requires tethering of HPV genomes to host mitotic chromatin via the viral E2 protein, enabling partitioning of replicated genomes into newly divided daughter cells. When basal cells begin the highly regulated process of epithelial differentiation, PV genomes are maintained in the differentiating cell. Finally, the viral genomes are amplified to a high copy number during the vegetative, or productive, stage of virus replication. Regulation of the switch from maintenance to vegetative PV DNA replication is incompletely understood but requires epithelial differentiation. The HPV E6 and E7 proteins are not themselves directly involved in DNA replication, but they promote cell cycle progression in the otherwise differentiating epithelial cells. This enables expression of the cellular machinery required for viral DNA replication.

HPV transcription occurs from two major promoters: an early promoter present in the ~1 kbp viral non-coding region (variously referred to as the long coding region (LCR) or the upstream regulatory region (URR)) and a downstream late promoter. These have been extensively characterized in HPV31, where they are present at nucleotide 97 (p97) and nucleotide 742 (p742), respectively. The corresponding sites in HPV16 are p97 and p670 and in HPV18 are p55 and p811 [3,12,13,14,15]. Transcription from the HPV genome is unidirectional and is controlled by several mechanisms that are each related to differentiation. HPV early and late promoter activity is regulated by differentiation, with relatively little early promoter activity in basal cells and a differentiation-dependent increase in late viral promoter activity following viral DNA replication [13]. In addition, viral transcripts are subject to numerous splicing events, some of which are regulated by differentiation. For high-risk HPV, E6 and E7 are transcribed as a bicistronic early transcript and splicing of E6 is required for E7 translation [16,17]. In contrast, low-risk genus alpha HPV and genus beta HPV transcribe E6 and E7 from two distinct promoters and do not exhibit the corresponding splice in E6 [18]. Another major splicing event is a differentiation-dependent splice that is required to produce the E1^E4 transcript encoding the E4 protein. Although it is encoded in the viral early region, E4 is expressed from the late RNA transcript following the onset of vegetative DNA replication and is important for virion production and infectivity [19,20]. In summary, the late stages of HPV replication are dependent on keratinocyte differentiation and the ways in which differentiation enables replication have been incompletely defined. Cellular differentiation is required for HPV replication, and intricate links between virus and host differentiation-dependent biology control many aspects of HPV replication. Although beyond the scope of this review, many aspects of differentiation-dependent HPV gene regulation have been studied and summarized comprehensively elsewhere [3].

## 3. Keratinocyte Differentiation and Models of HPV Replication

### 3.1. Stages of Keratinocyte Differentiation

Epithelial differentiation is a constantly occurring regenerative process that forms the protective exterior barrier between an organism and its surrounding environment. Best studied in mammalian skin, the process of epidermal differentiation originates in proliferative basal keratinocytes that are separated from the dermis by a specialized form of extracellular matrix (ECM), the basement membrane [21]. Laminins on the basement membrane attach to integrins, such as α6β4 and α3β1, on the basal cell surface to tether basal cells to the ECM. Adherens junctions connect basal cells to one another. Proliferative basal epithelial cells express keratin 5 and keratin 14.

**Figure 1:**
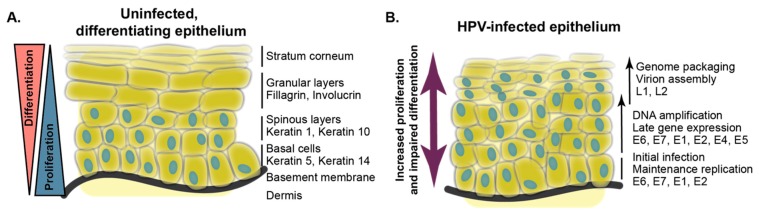
Human papillomavirus (HPV) replication requires and impairs epithelial differentiation. A schematic of epithelial differentiation in the absence (**A**) and presence (**B**) of HPV infection. In uninfected cells, self-renewing basal keratinocytes attach to the basement membrane. When keratin 5 and keratin 14-positive basal cells divide, one daughter cell can progress into the spinous layers and express keratins 1 and 10. Terminal differentiation and progression into the granular and cornified layers is marked by the expression of fillagrin and involucrin. Suprabasal epithelial cells are generally not proliferative. In an HPV-infected epithelium, proliferation is uncoupled from differentiation. Markers of proliferation are expressed and differentiation-related gene expression is impaired in suprabasal cells.

The first transition in the differentiation program is the delamination of a basal cell and its progression to a suprabasal (spinous) state [22,23]. Suprabasal keratinocytes express keratin 1 and keratin 10, which encode intermediate filament proteins that enable the formation of a cell–cell network connected by desmosomes. The next most distal layers to the ECM are the granular layer and the stratum corneum, respectively, and later markers of epithelial differentiation expressed in these layers include involucrin, loricrin, and fillagrin (Figure 1). By the time the keratinocyte differentiation program is complete, the once-proliferative cell exists as a squame, a dead cellular sac lacking a nucleus or other organelles. It is highly crosslinked and encased in a waterproof lipid network providing the exterior barrier function. Although many of the same cellular markers are expressed in the same order in mucosal versus cutaneous epithelia, it is important to note that differences in gene expression in these distinct tissues exist and enable their biological differences [24,25].

### 3.2. Differentiation Models

Several aspects of the differentiation-dependent HPV life cycle can be modeled in keratinocyte culture systems [26,27,28]. Human keratinocytes that harbor episomal (circular) and/or integrated HPV genomes have been both isolated from patient samples and generated experimentally. Keratinocytes can be induced to differentiate in monolayer culture using treatment with an increased concentration of calcium (typically 1.2–1.5 mM CaCl_2_), with or without the withdrawal of growth factors and/or the addition of serum. For many of the HPV-episome-containing cell lines, calcium treatment induces viral DNA amplification, transcription from the late promoter, and differentiation-dependent viral transcript splicing events. Culture in methylcellulose-based semisolid medium can promote many of the same changes. Three-dimensional cell culture systems (organotypic “raft” cultures) have been employed by HPV researchers for several decades. In such cultures, keratinocytes are grown to confluence on a matrix (often a collagen matrix containing mouse fibroblasts), then lifted to the air–liquid interface and allowed to stratify and differentiate for a period of 1–2 weeks. For cells that contain viral episomes, this allows for somewhat heterogenous differentiation and mimics some aspects of the viral life cycle. HPV virions are typically produced from organotypic cultures only rather inefficiently, especially in the case of immortalized cell lines. New PV model systems are sure to enable additional investigation. For example, a recently discovered murine papillomavirus has prompted additional studies on the relationship between cutaneous PV biology and epithelial differentiation [29,30,31,32,33,34].

Experiments in these systems have demonstrated that differentiation is required for HPV replication. Some specific cellular pathways that regulate epithelial differentiation have been found to regulate HPV genome amplification and late transcription. Protein kinase C (PKC) inhibitors limit HPV late activities, and efficient HPV virion production from organotypic raft cultures requires PKC activation [35,36]. Other experiments in keratinocyte differentiation models have begun to illustrate the effects of the HPV gene products on epithelial differentiation. These studies are reviewed in detail herein.

## 4. HPV Carcinogenic Activity and the E6 and E7 Oncoproteins

Just over one dozen mucosal HPV, primarily found in genus alpha species 7 and 9, are the “high-risk” HPV genotypes that cause nearly all cervical cancer and many other anogenital cancers [37,38]. High-risk HPV infection is responsible for approximately 5% of the worldwide cancer burden. In regions with limited access to screening and prevention programs, cervical cancer remains a major cause of mortality. Cancers of the oropharynx can be caused by high-risk HPV infection, predominantly by HPV16. In contrast to many other cancer types, the incidence of HPV-positive head and neck squamous cell carcinoma (HNSCC) is increasing in many populations, and in the United States alone there are now more cases of HPV-positive HNSCC than of cervical cancer [39]. Screening and prevention programs for HPV-positive oropharyngeal cancer and for HPV-associated genital cancers other than cervical cancer do not exist, nor are pharmacological approaches available to treat existing HPV infections. ‘Low-risk’ HPV, such as HPV6 and HPV11, cause lesions that rarely progress to cancer, but infection with low-risk HPV can cause genital warts, laryngeal papillomas, and other mucosal lesions [2]. Comparing high- to low-risk HPV has provided a wealth of information about the carcinogenic activity of the high-risk HPV proteins [40]. Many of the cellular reprogramming events required to enable HPV replication in the differentiating keratinocyte are likely to be conserved among diverse HPV types. Consequently, biological activities that are specific to high-risk HPV may account for their carcinogenic potential.

The contributions of the HPV E6 and E7 oncogenes to carcinogenesis have been studied extensively. A few years after zur Hausen and colleagues published the initial reports that cervical cancer samples contain human papillomavirus DNA, zur Hausen’s and other groups found that the HPV E6 and E7 ORFs are specifically preserved in cervical cancer cells and that they have transforming activity in rodent cells [41,42,43,44,45,46,47,48,49,50]. Further molecular analysis of the E6 and E7 gene products led to a model in which E7 proteins bind to retinoblastoma family proteins, including RB1, releasing E2F transcription factors and allowing passage through the G1/S checkpoint [51,52,53]. HPV E7 also promote proliferation by inhibiting the CDK inhibitors p21^WAF1/CIP1^ and p27^KIP1^ [54,55,56]. A consequence of high-risk HPV E7 expression is p53 stabilization [57]. To counter the apoptotic signaling that would otherwise be triggered by p53 stabilization, high-risk E6 proteins recruit the cellular ubiquitin ligase E6AP/UBE3A to enable p53 degradation [58,59]. In addition, E6 and E7 trigger genomic instability [60,61,62,63]. Oncogenic transformation by HPV results from persistent, long-term dysregulated expression of high-risk HPV E6 and E7 proteins [64,65].

This existing model does not account for all of the available information concerning the activities of HPV E6 and E7 [66]. Most HPV E7 proteins bind to RB1 and promote E2F-dependent gene expression, but HPV E7 proteins have varying ability to transform cells. Low-risk E7 proteins have very little transforming activity [67] and HPV1 E7 binds RB1 with high affinity but does not transform [68,69]. For several decades, HPV researchers have speculated that high-risk HPV E7 proteins have an RB1-independent transforming activity. This is supported by at least two lines of evidence. First, mutational analysis shows that alterations in the HPV16 E7 N- and C-termini result in a protein that is competent to bind RB1 but impaired for transformation [70,71,72,73,74,75]. Second, mouse models of cervical cancer and head and neck cancer provide evidence for an RB1-independent transforming activity of HPV E7 in vivo. In the oral model, <40% of mice develop tumors when RB1 is deleted, but the addition of HPV16 E7 to the RB1 knockout results in >90% tumor incidence [76]. In the cervical model, HPV16 E7 can induce cancer even when the transgenic mouse expresses an RB1 allele that cannot be bound by E7 [77,78]. Overall, there is not a consensus on which cellular targets might account for the RB1-independent transforming activity. Some recent findings address the initiation of carcinogenesis, accounting for the difference between high-risk and low-risk HPV E7 by demonstrating that high-risk HPV E7 upregulate the cell cycle inhibitors (CKI) p16^INK4A^/p14^ARF^ epigenetically [79,80,81]. RB1 degradation by high-risk HPV E7 is proposed to enable evasion of the oncogene-induced senescence (OIS) that would result from p16^INK4A^ induction.

A recent sequencing study highlights the critical contribution of HPV16 E7 to cervical carcinogenesis [82]. In this report, targeted sequencing of over 5000 patient samples from benign and precancerous HPV-positive cervical lesions revealed remarkable variability of HPV16 DNA sequences among patients. Depending on the cohort, in ~75–85% of patient samples the major HPV16 species detected had an HPV16 sequence unique among the thousands of patients. Furthermore, the variation in E6 was not the same as the variation in E7. In asymptomatic individuals, E6 and E7 sequences were both highly variable. In contrast, in precancerous lesions E6 sequences varied significantly, whereas E7 sequences were almost completely conserved. This suggests that if a lesion is to undergo malignant progression, there is a strong preference for the maintenance of the prototypical E7 sequence and that E7 is the high-risk HPV oncogene that is the major driver of oncogenic progression [82,83]. This result in human samples is consistent with the earlier finding that in a mouse model comparing HPV16 E6 and E7, it is E7 that is the dominant driver of estrogen-induced cervical carcinogenesis [84].

Updated models of oncogenic transformation by high-risk HPV have the potential to incorporate the new information provided by high-throughput studies and to account for activities of the viral oncoproteins that are not included in existing models. It is often stated that HPV oncoproteins act to uncouple proliferation from differentiation [4]. However, it is not clear whether HPV oncoproteins must impair differentiation to cause cancer and if so, what cellular targets are involved. Future studies should address whether the carcinogenic activity of specific HPV genotypes is related to their differentiation-inhibitory activities. An understanding of HPV-mediated carcinogenesis that includes regulation of differentiation might address some of the shortcomings of the existing model. If cellular targets required for HPV oncoproteins to modulate differentiation could be identified, these might provide tractable therapeutic targets.

## 5. HPV-Associated Cancer and Differentiation

The recognition that a subset of HNSCC are caused by HPV has enabled the comparison of HPV-positive to HPV-negative tumors at a similar anatomical site. A finding from pathology reports is that HPV-positive HNSCC tend to be poorly differentiated compared to HPV-negative HNSCC [85,86]. A recent analysis from my laboratory has demonstrated that this difference is also reflected by gene expression in HPV-positive relative to HPV-negative HNSCC [87]. Using data from The Cancer Genome Atlas (TCGA), we found that with respect to repressed genes, HPV-positive and HPV-negative cancers differ primarily in gene sets related to epithelial differentiation. Furthermore, we analyzed data from a comparison of primary human foreskin keratinocytes (HFK) to HFK expressing HPV16 E6 and E7 and found that the same downregulation of differentiation gene expression was dependent on the viral oncogenes [87,88]. Overall, these data indicate that HPV-positive cancers are poorly differentiated relative to HPV-negative cancers and that this is a consequence of E6/E7 expression.

Some previous studies in the pathology literature are consistent with this hypothesis even though they did not directly assess HPV status. A 1996 study observed a large range of involucrin expression in HNSCC and reported that poorly differentiated HNSCC are more likely to distantly metastasize [89]. Propensity for metastasis is a feature of HPV-positive HNSCC. A more recent analysis proposes that the relationship between HPV status and tumor differentiation is more pronounced in certain subtypes of HNSCC. Keck and colleagues described three subtypes of HNSCC: (i) a basal subtype that did not include any HPV-positive tumors and in which all the samples were well-keratinized and differentiated, (ii) an inflamed/mesenchymal subtype that includes some HPV-positive and some HPV-negative tumors and in which the HPV-positive cancers are nonkeratinizing and poorly differentiated, and (iii) a classical subtype in which HPV-positive and HPV-negative samples are not distinguished by differentiation status [90]. Keratin 19 appears to be expressed more predominantly in epithelial carcinomas compared to normal cells and it is highly expressed in HPV-positive compared to HPV-negative oropharyngeal cancers [91,92,93].

## 6. High-Risk HPV Oncoproteins Alter Epithelial Differentiation

### 6.1. Delay of Differentiation in Organotypic Culture

Soon after observing that high-risk HPV oncogene DNA was preserved in cervical cancer cells, researchers began to conduct studies on the oncogenes and on the entire HPV genome in differentiation models in keratinocytes. They observed that HPV DNA-positive HFK or SCC-13 cells could stratify in raft cultures and that the cells expressed keratins but did not differentiate normally. Hematoxylin and eosin staining on HPV-positive raft cultures indicates that the abnormal morphology resulting from HPV DNA is comparable to the abnormal morphology in a cervical or penile intraepithelial neoplasia [94]. Initial studies on HPV16 were soon extended to include HPV18. In normal epithelium, proliferative cells are restricted to the basal epithelial layers, but in raft cultures expressing both HPV18 E6 and E7 or the complete HPV18 genome, parabasal cells extend throughout the stratified epithelium [94,95,96,97,98]. More recent studies in organotypic raft cultures have examined additional HPV types. A comparison of nine established high-risk HPV types and two probable high-risk HPV types by Schutze and colleagues determined that all of these viruses alter differentiation in an organotypic raft culture as assessed by keratin 10 staining [99].

Researchers have examined high-risk HPV E6 or E7 alone and/or used mutant genomes that do not express E6 or E7 in order to assess the individual contributions of the oncoproteins. The strongest effects on differentiation occur when E6 and E7 are expressed together. Organotypic cultures that express high-risk HPV E7 consistently exhibit an increase in proliferation in suprabasal cells. Several of the initial reports concluded that high-risk HPV E7 has more differentiation-inhibiting activity than HPV E6 [62,96,97,100,101]. This was confirmed by recent work using organotypic cultures of HaCat cells in which intact HPV16 genomes promote hyperproliferation and partially inhibit differentiation [102]. Cells harboring mutant genomes that did not express HPV16 E7 formed rafts in which keratin 14 expression was not restricted to basal cells and in which involucrin expression was increased. Compared to HPV16 E6, HPV16 E7 had the stronger effect on promoting hyperproliferation and disturbing normal epithelial differentiation. However, high-risk HPV E6 also exhibit differentiation inhibitory activity. This appears to predominate in basal cells, but in some cases HPV16 E6 can impair differentiation in organotypic cultures [103,104]. Relatively few studies have assessed low-risk HPV oncogenes in these assays, but those that have suggest that low-risk HPV E7 have less ability to impair differentiation than high-risk HPV E7 [62,96]. Some mutational analysis has assessed which activity of high-risk HPV E7 might mediate its effects on differentiation (see Section 6.4).

### 6.2. Downregulation of Differentiation-Related Cellular Gene Expression

Transcriptional studies on normal and HPV-DNA-containing cells and on HPV-positive and negative cancer cells illustrate that HPV gene expression impairs epithelial differentiation. Consistent with the analysis of raft cultures, human cells harboring high-risk HPV genomes express lower levels of differentiation marker genes and both high-risk HPV E6 and E7 likely contribute to this repression. Most work has focused on the HPV16 oncogenes, with some analysis of HPV18 and minimal examination of low-risk HPV. Early cDNA array experiments examined a few thousand cellular genes and compared normal cells to cells expressing HPV16 E6 and E7 [105]. The strongest effects of the oncoproteins on gene expression were in cells induced to differentiate via growth factor withdrawal and calcium treatment. When the oncogenes were analyzed separately, introducing HPV16 E7 downregulated some markers of keratinocyte differentiation and further introduction of HPV16 E6 augmented the downregulation some of these markers and moderated the repression of others [106].

Additional transcriptional studies on HPV16 immortalized cell lines and on cervical cancer cell lines demonstrate that the repression of differentiation is maintained over time in HPV-positive cells. Wan and colleagues compared normal cells to early passage HPV16-immortalized and to late passage HPV16-immortalized cells and noted that downregulation of epithelial structural genes is reflected both in the early and late cell models [107]. These authors also found that some HPV-mediated changes in keratin expression were reflected in an immunohistochemistry database containing HPV16-positive samples. In a subsequent study, comparing HPV16- and HPV18-positive cervical cancer cells to normal cells indicated that several hundred genes were up- or downregulated in the cancer cells. The downregulated genes included differentiation-related transcripts such as serine protease inhibitors, TGFβ1, and keratin 16 [108]. Advances in microarray technology enabled the analysis of more transcripts and patient samples. A 2006 study analyzed 29 cervical cancer samples, most of them HPV16-positive, and 18 normal controls, again finding that genes downregulated in cervical cancer included many markers of epithelial differentiation plus other genes related to immune signaling [109]. The downregulated genes related to keratinocyte differentiation included keratins, involucrin, serine proteases and protease inhibitors, and desmoglein 1. A general limitation of these early microarray studies is that the ability to detect transcripts was not matched by computational pathway analysis tools.

More recent analyses have combined transcriptional data with pathway analysis. Overall, pathways related to cell cycle, cellular assembly and organization, DNA replication, and growth and proliferation are dysregulated by HPV16 E6 and E7. Basal keratins, such as keratin 5, are relatively unaffected by HPV16 E6 and E7, but HPV16 E7 tends to downregulate individual genes related to epithelial differentiation and a similar effect is observed with HPV16 E6 alone or with HPV16 E6 and E7 expressed together [110]. Recently, Klymenko and colleagues used RNA sequencing and pathway analysis to compare the response to calcium-mediated differentiation in normal immortalized keratinocytes (NIKS) versus NIKS harboring episomal HPV16 genomes (NIKS-16) [111]. Many downregulated genes in this analysis are related to epithelial barrier function, cytoskeleton, and cell adhesion, indicating that HPV16 is able to repress the epithelial differentiation program even in the presence of a strong differentiation-promoting signal. Differentiated cells harboring HPV16 DNA also exhibit a gene expression signature reflecting downregulation of immune-function-related pathways. In this and in other studies, HPV gene expression appears to have the strongest effects on relatively early markers of epithelial differentiation, such as keratin 10, compared to a more modest downregulation of expression of late differentiation proteins such as involucrin. At least one study has examined the effects of HPV16 E6 and E7 on an involucrin promoter–luciferase reporter construct with similar results [112].

### 6.3. HPV E6 and Differentiation

#### 6.3.1. MAML1, Notch, and Genus Beta HPV E6

Genus beta HPV infect the cutaneous epithelium. A subset of beta HPV cause hyperproliferative lesions in the skin of patients with the genetic disorder Epidermodysplasia Verruciformis, and links between beta HPV and skin cancer have also been proposed [113]. Several recent studies have defined the cellular binding partners of the genus beta versus genus alpha HPV oncoproteins. Somewhat unexpectedly, these efforts revealed that the cellular proteins bound by genus beta HPV E6 proteins are distinct from those targeted by genus alpha HPV E6 [114,115]. Two large-scale proteomics efforts identified MAML1 as a binding partner of genus beta HPV E6. Rozenblatt-Rosen and colleagues identified HPV5 and HPV8 E6 in complex with MAML1 [115]. Our own analysis of eight beta HPV E6 and eight genus alpha HPV E6 confirmed that the beta HPV E6–MAML1 interaction is conserved across HPV genus beta and not shared by genus alpha HPV E6 [114]. Subsequent analyses found that the bovine papillomavirus type 1 (BPV1) E6 protein also binds to MAML1 [116,117]. BPV1 E6 has activity in transformation assays but does not engage p53, and so the molecular basis of its transforming activity has been the subject of some investigation [118,119,120,121]. Although some genus beta HPV E6 bind and/or stabilize p53, this is not conserved among genus beta HPV E6 and the effect on p53 transcriptional activity varies among the beta HPV E6 [122]. Consequently, the identification of MAML1 as a conserved interactor of genus beta HPV E6 sparked interest for its potential to explain biological properties of the cutaneous HPV.

Elegant structural studies on papillomavirus E6 proteins demonstrate how HPV E6 achieve specific binding to their cellular targets [123,124,125]. PV E6 engage several of their cellular targets by binding to an acidic leucine-containing motif (‘LxxLL’) in the cellular protein. Cellular proteins that contain the LxxLL motif and that are targeted by one or more PV E6 proteins include E6AP, MAML1, and paxillin [120,121,126,127,128,129]. The LxxLL peptide is bound by a hydrophobic pocket in E6 formed by two zinc-binding domains separated by an alpha-helical linker. Differences in the residues that form the pocket in E6 confer specificity for a particular LxxLL motif [125]. This model has been validated by an extraordinarily comprehensive analysis of diverse human and animal papillomavirus E6 proteins [130]. Most PV E6 tested in that study bind MAML1 and inhibit a GAL-MAML1 transcriptional reporter, whereas some genus alpha HPV E6 bind E6AP.

Several signaling pathways are affected by MAML1, including those involving Notch, p53, MEF2C, and beta-catenin [131,132]. However, the predominant effect of beta HPV E6 binding to MAML1 appears to be on NOTCH signaling. NOTCH was first characterized as an oncogene in T cells, but it is a tumor suppressor in the skin where it serves to promote differentiation [133,134]. NOTCH1-4 comprise a family of single-pass transmembrane proteins that are activated upon interaction with a Delta-like or Jagged ligand. Such interaction and subsequent cleavage of NOTCH by metalloprotease and gamma-secretase allows for the release of the NOTCH intracellular domain (ICD) and its translocation to the nucleus. In the nucleus, the NOTCH ICD forms a complex with CSL DNA-binding proteins (CSL: CBF1/RBP-J (mammalian), Su(H) (Drosophila), and Lag-1 (Caenorhabditis elegans)), displacing the repressors of CSL activity and allowing for the recruitment of transcriptional co-activators. RBP-J/CBF1 is the mammalian repressor and MAML1 is one such co-activator [131,135,136]. The HPV proteomics experiments and further validation studies indicate that the beta HPV E6–MAML1 complex can also include RBP-J and the NOTCH ICD [32,114,116,137].

**Figure 2:**
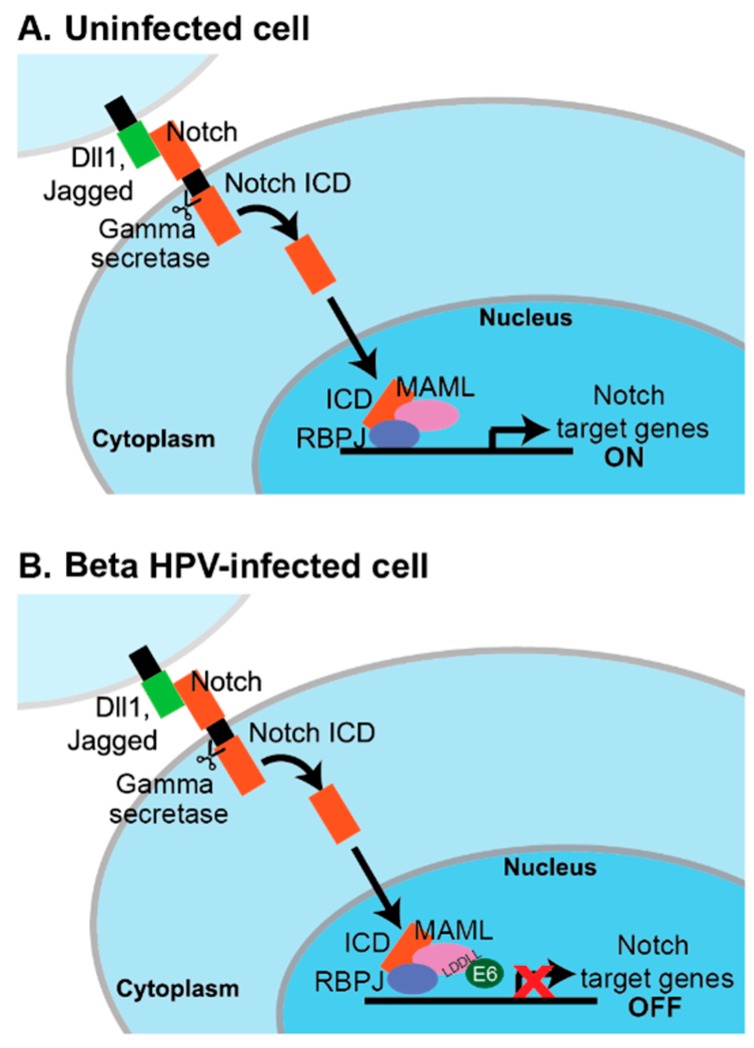
Genus beta HPV E6 bind MAML1 to inhibit NOTCH signaling. A schematic of activated NOTCH signaling in the absence (**A**) and presence (**B**) of genus beta HPV E6. Upon activation of the NOTCH signaling pathway, proteolytic cleavage of NOTCH releases the NOTCH intracellular domain (ICD), which can translocate to the nucleus. It displaces transcriptional repressors and forms a transcriptionally active complex including NOTCH, MAML1, and RBP-J. Genus beta HPV E6 bind to an LxxLL motif present in the MAML1 C-terminus. The oncoprotein does not disrupt formation of the transcription complex but does inhibit NOTCH signaling.

Several groups used NOTCH-MAML1 reporter assays and analyzed endogenous NOTCH-responsive transcripts to find that beta HPV E6 inhibit signaling in response to a NOTCH stimulus [116,117,137]. The LxxLL motif bound by beta HPV E6 proteins is at the C-terminus of MAML1, which is the region important for its transcriptional co-activator activity [138]. The working model is that beta HPV E6 do not impair the formation of the MAML1–RBP-J–ICN complex at NOTCH-responsive promoters, but block the recruitment of additional components required for transcription [137] (Figure 2). Some details of this model remain to be tested. For example, CBP and EP300 can be co-activating components of a MAML1–NOTCH signaling complex, but only a small subset of beta HPV E6 bind to CBP and EP300, whereas a large number of E6 bind to MAML1 and inhibit a MAML1-dependent transcriptional reporter [114,130]. Proteins encoded by other viruses, including Epstein–Barr virus (EBV), Kaposi’s Sarcoma Herpesvirus (KSHV), and adenovirus, are known to interact with the Notch/RBP-J complex and to regulate transcription from both viral and host cellular promoters [139,140,141,142,143,144,145].

These results have been interpreted to mean that beta HPV E6 proteins bind MAML1 to repress epithelial differentiation. Presumably, this would enable maintenance of a more proliferative state in the beta HPV infected epithelial cell and a cellular environment that promotes persistence and viral DNA replication. Meyers and colleagues demonstrated that HPV8 E6 significantly inhibits the calcium-responsive transcription of HES1 and the calcium-dependent inhibition of involucrin in keratinocytes [137]. This inhibition occurs to a comparable degree as inhibiting NOTCH cleavage with a gamma-secretase inhibitor, but the use of a Notch pathway targeted transcriptional array suggests that the effects of HPV8 E6 are not identical to the effects of chemical gamma-secretase inhibition.

The lack of robust life cycle models for the genus beta HPV means that these predictions regarding differentiation have not been tested in organotypic cultures. However, a recently discovered mouse papillomavirus, MmuPV1, shows promise as a model of cutaneous PV biology [29]. MmuPV1 E6 binds to MAML1 and inhibits Notch signaling. Single amino acid changes in MmuPV1 E6 impair binding to MAML1 and limit the ability of MmuPV1 to cause papillomas in nude mice [32]. The interaction of cutaneous PV E6 proteins with MAML1 contributes to the ability of these E6 to impair epithelial differentiation. The ability to test this hypothesis in an animal model will further elucidate the relationship between differentiation inhibition and papillomavirus-associated disease.

#### 6.3.2. Genus Alpha HPV E6

The effects of genus alpha HPV E6 on differentiation and the mechanisms by which they might act are less well established than for genus beta HPV E6. A differentiation-inhibitory effect of HPV16 E6 was identified in early studies that measured the ability of oncoproteins to induce resistance to calcium-mediated differentiation [146]. Later versions of this assay demonstrated some activity for both E6 and E7, but indicated that they acted more efficiently together [147]. The transcriptional studies already introduced suggest that HPV16 E6 downregulates the transcription of genes related to epithelial differentiation, particularly in monolayer culture. Variants of HPV16 E6 are mostly impaired with respect to their ability to restrict differentiation [104,148], although now with the advent of high-quality structures for HPV16 E6 it may be helpful to reassess whether such variants are stably expressed and able to engage their cellular targets.

Like genus beta HPV E6, HPV16 E6 has been suggested to alter Notch signaling, although the underlying mechanism(s) are less clear than for the cutaneous HPV E6. MAML1 was initially identified in a yeast-two-hybrid screen for binding partners of HPV16 E6 [138], but the HPV16 E6–MAML1 interaction was not subsequently observed in any of the recent proteomic analyses of HPV E6 [114,115]. It has been proposed that HPV16 E6 inhibits NOTCH dependent on degradation of p53 or dependent on TAp63β [149,150,151,152]. Kranjec and colleagues recently reported that HPV16 E6 promotes proliferation of high-density keratinocyte cultures and that it acts primarily in basal cells through the combined inactivation of p53 and NOTCH [153]. They propose that HPV16 E6 inhibits NOTCH cleavage and decreases NOTCH transcription dependent on p53 degradation, although the details of such a mechanism remain to be elucidated.

#### 6.3.3. Notch and SCC

Inactivation of the NOTCH signaling pathway by cutaneous HPV is consistent with the tumor-suppressive role of NOTCH in the skin [154,155]. NOTCH signaling is activated in epithelial cells that have divided and left the basal layer, and this occurs concomitant with or dependent on a decrease in p63 levels [144]. The specific NOTCH target genes responsible for induction of the differentiation program remain to be determined.

Studies in cervical cancer cell lines indicate that NOTCH signaling is dysregulated in cervical cancer, although there is not yet a consensus on whether manipulating NOTCH signaling in these cells promotes or inhibits cancer cell proliferation [156,157,158,159,160,161]. The finding that genes in the NOTCH pathway are frequently mutated in HNSCC led to the conclusion that NOTCH is tumor suppressive in HNSCC [162,163]. As it is in cervical cancer, the actual relationship may be more complex. A variety of NOTCH mutations have now been observed in various populations of HNSCC patients, some activating and some inactivating [164]. A recent study examined the differences in NOTCH1 expression in HPV-positive versus HPV-negative HNSCC and concluded that there is an association between NOTCH1 mutation and HPV status. Specifically, tumors wild-type for NOTCH1 are more likely to be HPV-positive than NOTCH1-mutated tumors [165]. More investigation will be required to clarify whether high-risk HPV oncoproteins inactivate NOTCH signaling.

#### 6.3.4. TGFβ

In addition to NOTCH signaling, other pathways impact epithelial differentiation and are affected by HPV E6. TGFβ signaling begins with the TGFβ ligand binding to serine/threonine kinase receptor complexes. The intracellular binding partners of the TGFβ receptor are receptor-associated SMAD (R-SMAD) and co-activator SMAD (co-SMAD) proteins. Phosphorylation events allow for the assembly of an R-SMAD/co-SMAD complex on target gene promoters. The complex can transactivate the promoters of target genes, such as p21^CIP1^ (CDKN1A), p27^KIP1^ (CDKN1B), and p15^INK4B^ (CDKN2B). The net effect of activation of these cell cycle inhibitors is cell cycle arrest in G_1_. TGFβ signaling controls epithelial differentiation and can have context-dependent tumor suppressive or oncogenic activities. The pathway is frequently mutated in epithelial tumors such that its activity is lost [166,167,168,169], but its activation can also promote the epithelial–mesenchymal transition and thus drive invasion [170].

One of the early transcriptional studies on gene expression changes induced by HPV16 E6 and E7 noted that the viral oncoproteins alter the cellular response to TGFβ [105]. The authors of this study found overlap between genes induced by the viral oncoproteins and genes induced by TGFβ and proposed that HPV16 E6 and E7 altered expression of TGFβ and TGFβ-inducible genes as a result of inhibiting differentiation. Consistent with this, co-expression of HPV16 E6 and E7 decreased secretion of TGFβ2 after treatment with the differentiation-inducing agent all-*trans* retinoic acid (ATRA).

The mechanistic basis for effects on TGFβ signaling has been more clearly established for the genus beta HPV E6 proteins. Mendoza and colleagues found in a yeast-two-hybrid experiment that HPV5 E6, but not HPV9 E6, bound to SMAD3 [171]. HPV5 E6 was consequently able to repress TGFβ signaling by binding SMAD3 and perhaps to destabilize both SMAD3 and SMAD4. HPV5 E6 but not HPV5 E7 was able to repress a SMAD-dependent luciferase reporter, indicating that this effect is specific for certain HPV oncoproteins.

More recent experiments have further elucidated the interaction between genus beta HPV E6 and TGFβ signaling. The HPV E6 proteomics experiments identified several genus beta HPV E6 in complex with SMAD2 and SMAD3 [114,115]. Meyers and colleagues went on to determine that beta HPV E6 oncoproteins do not alter the TGFβ-dependent changes in SMAD2/3 phosphorylation nor do they affect SMAD2/3 nuclear localization [32]. The beta HPV E6 proteins do appear to interfere with the formation of the SMAD-containing transcriptional complex at the p15^INK4B^ (CDKN2B) promoter after TGFβ treatment.

### 6.4. HPV E7 and Differentiation

#### 6.4.1. Inhibition of Differentiation by HPV E7

The pro-proliferative effect of HPV E7 has been documented extensively [172]. This section will highlight the evidence that HPV E7 can delay or impair cellular differentiation. Although increased proliferation could indirectly restrict differentiation, there is evidence for direct effects of HPV E7 on the epithelial differentiation program independent of effects on proliferation. This is supported by work in organotypic culture, in monolayer cells, and in mouse models.

Epithelial differentiation in organotypic culture is impaired by episomal HPV16 DNA dependent on the HPV16 E7 ORF [95,100]. A recent study directly compared E5, E6, or E7-null HPV16 genomes and found that although loss of any one of the three oncoproteins alters epithelial morphology, the HPV-positive rafts lacking E7 were particularly impaired [102]. HPV16 E7-null rafts exhibit a striking loss of keratin 14 staining in suprabasal layers. Further analysis of HPV16 E7 mutants indicates that the induction of suprabasal DNA synthesis by HPV16 E7 is separate from its ability to perturb differentiation [173]. HPV16 E7 ∆PTLHE is altered in conserved region 1 (CR1) and can bind to but not degrade the pocket proteins RB1, RBL1/p107, and RBL2/p130. It is particularly impaired in limiting differentiation in raft cultures, leading to the conclusion that pocket protein degradation is required for the inhibition of differentiation but not for HPV16 E7 to promote suprabasal DNA synthesis [173]. Several cutaneous HPV E7 also exhibit evidence of differentiation inhibition in organotypic culture [174].

In monolayer culture, HPV16-E7-expressing cells resist differentiation induced by calcium treatment [56]. p21^CIP1^ links differentiation and proliferation in keratinocytes and the differentiation-inhibitory effect of HPV16 E7 has been ascribed to its ability to bind to p21^CIP1^ and block the inactivation of cyclin/cdk complexes [54,56]. In a monolayer culture assay that measures the ability of HPV16 E7 to inhibit myoblast differentiation, HPV16 E7 decreases expression of a differentiation marker and both HPV16 E7 ∆PTLHE and RB1-binding domain mutants are impaired in this activity [72]. Low-risk mucosal HPV E7 proteins have been examined less frequently for their ability to alter differentiation, but at least one study reported that HPV6 E7 can reduce involucrin protein expression after calcium treatment [175].

Several experiments in mice indicate that HPV16 E7 inhibits differentiation and suggest that this is independent of RB1 binding [78,176]. Balsitis and colleagues developed a mouse model to separate the RB1-dependent and RB1-independent activities of HPV16 E7. They used transgenic mice that express HPV16 E7 under the control of the keratin 14 promoter, then crossed them to mice harboring a mutant RB1 that cannot bind to HPV E7. Strikingly, in this model, even when HPV16 E7 cannot bind to RB1, it can still delay terminal differentiation and induce hyperplasia. This indicates that the ability of HPV16 E7 to delay or restrict differentiation is at least partially independent of its ability to bind RB1. A similar study in the mouse cervix emphasizes the importance of RB1-independent effects of HPV16 E7 in the mucosal epithelium [77].

#### 6.4.2. Differentiation Pathways Altered by HPV E7

Some cellular pathways related to differentiation are known to be dysregulated by HPV E7. HPV16 E7 can abrogate some effects of TGFβ treatment, such as TGFβ-mediated inhibition of the *myc* promoter [177]. Studies on TGFβ also highlight the differences between mucosal and cutaneous HPV oncoproteins. The SMAD-TGFβ reporter that was repressed by genus beta HPV E6 but not beta HPV E7 is affected differently by high-risk HPV16 oncoproteins [171]. Specifically, it was repressed by high-risk HPV E7 but not high-risk HPV E6. There is at least one report that HPV16 E7 can block binding of SMAD3 to its target sequence [178], but it should be noted that HPV E7 did not bind to SMAD proteins in any of the recent proteomic studies.

High-risk HPV E7 modulate levels of p63, a master regulator of epidermal development in epithelial cells [179]. An upstream regulator of p63 is miR-203, which increases in abundance as keratinocytes differentiate. p63 protein levels decrease upon epithelial differentiation, but in the presence of HPV31 genomes or HPV31 E7, miR-203 expression decreases and p63 levels are higher than in control cells [180]. p63 is required for HPV31 late functions, such as genome amplification and late viral gene expression. In HPV-positive keratinocytes depleted of p63, the levels of cell cycle regulatory proteins, such as cyclin A, cyclin B1, cdk1, and cdc25c, were reduced and late viral activities were impaired [181]. p63 protein levels are elevated in cervical cancer cell lines, dependent on HPV E7, and there is a good correlation between high p63 staining and HPV positivity in cervical lesions [182,183].

#### 6.4.3. HPV E7 and PTPN14

The mutational analyses of HPV16 E7 suggest that its differentiation-inhibitory activity could be independent of RB1 binding, but the RB1-independent activities of high-risk HPV have remained poorly defined. The proteomic analyses published by several groups have suggested a new RB1-independent activity of high-risk HPV E7 [114,115]. Diverse HPV E7 bind to the putative tumor suppressor PTPN14 and high-risk HPV E7 target PTPN14 for proteasome-mediated degradation using the ubiquitin ligase UBR4 [184,185]. The E7/PTPN14/UBR4 complex is distinct from the E7/RB1 complex and PTPN14 degradation correlates with the oncogenic activity of papillomavirus E7. The HPV16 E7 N-terminus is required for binding to the ubiquitin ligase UBR4 and targeting of the putative tumor suppressor PTPN14 for degradation [185].

Our recent work on PTPN14 and high-risk HPV E7 led us to discover that the loss of PTPN14 from human keratinocytes results in a striking downregulation of the epithelial differentiation program [87]. An N-terminal variant of HPV16 E7 that cannot degrade PTPN14 is impaired in keratinocyte immortalization and in restricting epithelial differentiation. We hypothesize that this finding could be related to the earlier work that described a defect in differentiation inhibition for an N-terminal HPV16 E7 deletion mutant. We propose that degradation of PTPN14 represents a critical RB1-independent function of high-risk HPV E7, that the effect of PTPN14 degradation is to limit differentiation, and that activity is essential for HPV-mediated oncogenic transformation.

Mechanistically, we do not yet understand how PTPN14 degradation could limit epithelial differentiation. PTPN14 is a non-receptor protein tyrosine phosphatase that negatively regulates the transcriptional co-activator YAP1 [186,187,188,189,190]. HPV E7 does not repress the canonical YAP1 targets CTGF and CYR61 as a result of PTPN14 degradation in human keratinocytes [87]. However, YAP1 has been reported to repress epithelial differentiation [191] and future experiments could assess whether this process is impacted by the high-risk HPV E7-mediated degradation of PTPN14.

### 6.5. HPV E5 and Differentiation

Most effort to understand how HPV manipulates epithelial differentiation has focused on the E6 and E7 oncogenes and on signaling pathways, including Notch and TGFβ. Additional HPV-encoded proteins likely impact differentiation and additional differentiation-related signaling pathways are altered during HPV infection. For example, a recent study comparing HPV18 genomes to HPV18 genomes that do not express the E5 protein found that E5 contributes to a repression of involucrin expression upon calcium-mediated differentiation [192].

## 7. Differentiation Therapy

Differentiation therapy, the induction of differentiation by a chemical agent, has been extremely successful in specific instances in the clinic. For example, acute promyelocytic leukemia can often be cured by treatment with ATRA and arsenic [193,194]. Although the mechanism underlying this cure is now recognized to be more complicated than initial reports suggested, the promise of differentiation induction as a relatively non-toxic therapeutic approach remains high [195]. ATRA has also shown promise in mouse models of non-melanoma skin cancer and in reducing proliferation of human squamous cell carcinoma cells [196,197,198]. HPV-positive cervical lesions, particularly lower-grade lesions, have shown some response to treatment with ATRA and other studies support the idea that ATRA could inhibit the growth of HPV-positive cell lines and/or tumors [199,200,201,202,203]. Further investigation of the role of differentiation therapy in treating HPV infections and HPV-associated cancers is important. No currently available therapeutic agents can treat the millions of new HPV infections that result in hundreds of thousands of new premalignant lesions and HPV-positive cancers each year. Disrupting any essential step in the carcinogenic progression of an HPV-positive lesion would have therapeutic potential, and an inhibition of differentiation by HPV oncoproteins may be such an essential step.

## 8. Conclusions and Open Questions

HPV-encoded proteins reprogram host cells to promote virus replication and enable persistent infection. In particular, HPV E6 and E7 drive the establishment of a pro-proliferative environment in the infected cell. They promote cell cycle re-entry in differentiating keratinocytes and restrict the epithelial differentiation program. Years of research on HPV E6 and E7 have enabled groundbreaking discoveries on the mechanisms that normally control the cell cycle and on how cell growth control pathways are dysregulated in cancer.

HPV E6 and E7 lack enzymatic activity, meaning that their reprogramming activities are enabled by interactions with host cellular proteins. Unbiased proteomic analyses have begun to reveal the host cellular targets that are shared among and/or unique to a subset of the HPV oncoproteins. Some of these targets appear to be used by the HPV oncoproteins to manipulate the differentiation program. For example, the genus beta HPV E6 proteins engage MAML1 and SMAD to modulate signaling in the NOTCH and TGFβ pathways [32,116,117,166]. We now understand that there are several mechanisms used by cutaneous HPV E6 to inhibit keratinocyte differentiation that are not shared by the genus alpha mucosal HPV.

Studies on the mucosal HPV oncoproteins indicate that both high-risk HPV E6 and E7 have differentiation inhibitory activity. The accumulated evidence suggests that high-risk HPV E7 have a stronger inhibitory effect on differentiation relative to high-risk HPV E6, particularly in three-dimensional culture models. Furthermore, some mutational analyses are consistent with the idea that high-risk HPV E7 has a differentiation-inhibitory effect that is not dependent on its ability to bind RB1. PTPN14 is one host cell target of high-risk HPV E7 that is targeted independent of RB1 and shows early promise as a regulator of keratinocyte differentiation, but more work to understand how high-risk HPV repress differentiation is warranted. In addition, most of the work on how genus alpha HPV oncoproteins affect differentiation has been on the high-risk HPV. It remains unclear whether high-risk HPV oncoproteins, particularly high-risk HPV E7, have a greater capacity to decrease differentiation than their low-risk counterparts. An additional challenge to directly comparing high-risk HPV E6 and E7 comes from the fact that in cells that contain the HPV genome, E7-encoding transcripts are more abundant than E6-encoding transcripts [16,17]. A direct comparison of the differentiation-inhibitory activities of the two proteins would require that they are expressed at equal levels. HPV E6 and E7 also regulate miRNAs, which could themselves impact keratinocyte differentiation [88,204].

Taken together, the results summarized in this review support the idea that cutaneous HPV E6 could have a greater differentiation-inhibitory capacity than cutaneous HPV E7, whereas mucosal HPV E7 is the more potent inhibitor of differentiation compared to mucosal E6. This hypothesis should be tested rigorously. Others have noted that when comparing HPV E6 and E7, E6 is the more potent oncoprotein in the skin whereas E7 is more potent in the mucosal epithelium [40]. It will be interesting to determine whether this difference is related to the ability of the oncoproteins to limit epithelial differentiation and to consider whether the distinct differentiation-related targets of the cutaneous versus mucosal HPV oncoproteins represent their having evolved to target biological differences in the tissues.

A final open question is whether inhibition of differentiation is required for HPV-mediated oncogenic transformation. It would seem logical that all HPV, not only high-risk HPV, must limit differentiation to enable viral genome replication in the stratified epithelium. However, differentiation is not only a normal process occurring in stratified epithelia, it is also a keratinocyte stress response. Oncogene-induced differentiation (OID) is the process by which keratinocytes differentiate in response to tumor suppressor (including p53) inactivation [205,206] or oncogene overexpression [207,208,209,210,211]. The importance of OID is emphasized by the observation that epithelial cancers frequently have mutated p53 but that p53 mutation is rarely sufficient to drive skin cancer [212,213]. Future studies should investigate whether high-risk HPV oncoproteins trigger differentiation as a result of tumor suppressor inactivation and if so, whether the differentiation-inhibitory activities of the oncoproteins are required to evade OID.
